# A Case of Chronic Cystitis and Urethritis with Ulcers

**Published:** 1894-02

**Authors:** C. D. Hurt

**Affiliations:** Atlanta, Ga.


### A CASE OF CHRONIC CYSTITIS AND URETHRITIS WITH ULCERS.*

BY C. D. HURT, M. D.,
ATLANTA, GA.

Mrs. ———, age twenty-seven, married second time, has lived with her present husband six years, has borne no children. Catamenial periods have been regular, though with considerable pain. Her bladder trouble began seven years since. At first she experienced pain in emptying the bladder and slight increase of frequency in desire. Later, burning pain while passing water beginning in the urethra and extending to the bladder.

She sought relief by applying to her family physician. Later, she went abroad and was under treatment for some months, with no

*Read before the Atlanta Obstetric Society, December 14th, 1893.

abatement of symptoms. She states that for the past four years she has had to empty her bladder every twenty to forty minutes, and usually more frequently at night. She has had to abandon all social pleasure, visiting and church going because of the frequent spasms of the bladder and the resistless desire to empty it. When called to see her, and while getting a history of her case, she felt impelled to empty her bladder. I watched her agonizing and twisting while endeavoring to do so and asked an inspection of the deposit. From one-half to two ounces of urine usually passed at a time, highly colored, with occasional blood clots and mucous shreds. The urine contained exfoliations from the lining of the bladder. Specific gravity, 1016.

I found the meatus swollen and red, everted and slit. She states that four years prior, two reputable physicians in treating her, had anesthetized her and introduced some instrument which lacerated the meatus. I introduced first a No. 7 soft rubber catheter, found the urethra extremely sensitive and the patient cried out with excruciating pain. I then injected into the bladder one-half ounce of warm solution of borax, grains thirty to four ounces of water. This was allowed to flow back through the catheter after being retained two minutes. Withdrawing the catheter, I used a twenty per cent. solution of cocaine and then introduced a No. 10 bougie, found two inches from the meatus and one-half inch from the bladder along the track of the urethra, two rough constrictions which proved to be deep ulcers. I introduced a straight silver catheter until the fenestra was directly over the ulcer, and then introduced through the catheter a mop made of absorbent cotton saturated with a solution of argenti nitras, grains fifteen to an ounce of water.

To be sure when the fenestra reached the ulcer, I had, on a previous examination, marked on the catheter its depth when in position, and was careful to have it properly adjusted. This operation repeated every fourth day, and in the interval injected once daily a solution of borax, increasing the quantity until the bladder would receive and hold without complaint four ounces.

Improvement was uninterrupted and she was dismissed at the end of four weeks with healthy bladder and urethra and normal functions.

As to the origin of the trouble from which this woman was suffering I have not fully decided in my own mind. I neglected to state that she had slight anteflexion of uterus with constriction of the neck. On account of this mal-position and deformity there had been no conception, and, in my opinion, it was the irritation from these causes that originated first her bladder trouble. I am of the opinion also that neglect of early treatment had something to do with creating this inflammatory condition of the bladder. This woman seemed in every respect healthy with the above exception. Habits of her bowels regular, menstruation was normal in every respect except being attended with an undue amount of pain. I had the oversight of her for twelve months after treating her and there was no return of her bladder trouble. I advised her to have local treatment for the relief of the stricture of the neck of the womb. This she declined on account of limited means.

We all know that catarrh of the bladder must have primarily some exciting cause, whether it be stone in the bladder or specific inflammation extending from the urethra, or mal-position of the uterus, or medicines taken internally which are calculated to have this effect.

